# CardioFM: A Multimodal Foundation Model for Joint ECG and PPG Representation Learning

**DOI:** 10.21203/rs.3.rs-9652631/v1

**Published:** 2026-05-11

**Authors:** Md Hassanuzzaman, Tilendra Choudhary, Alasdair Gent, Mihai V. Podgoreanu, Suresh Mitu Agarwal, Vijay Krishnamoorthy, Sivasubramanium Bhavani, Philip Yang, Annette Esper, Rishikesan Kamaleswaran

**Affiliations:** 1 Department of Electrical and Computer Engineering, Duke University, Durham, NC 27708, USA; 2 Department of Surgery, Duke University School of Medicine, Durham, NC 27710, USA; 3 Department of Anesthesiology, Duke University School of Medicine, Durham, NC 27710, USA; 4 Department of Medicine, Emory University, Atlanta, GA 30322, USA; 5 Division of Pulmonary, Allergy, Critical Care, and Sleep Medicine, Emory University, Atlanta, GA 30322, USA; 6 Department of Biomedical Engineering, Duke University, Durham, NC 27708, USA

## Abstract

Electrocardiography (ECG) and photoplethysmography (PPG) arise from the same heartbeat and are routinely co-acquired at every monitored bedside, yet no foundation model jointly encodes both modalities. Existing approaches are either ECG-specific, PPG-specific, or domain-agnostic, and none captures the cross-modal physiological coupling between cardiac electrical activity and peripheral hemodynamics. We present CardioFM, a self-supervised multimodal foundation model that integrates ECG Lead-II and PPG through bidirectional cross-modal attention and adaptive residual vector quantization. CardioFM is pretrained on over 500,000 hours from approximately 63,000 patients across intensive care, surgical, ambulatory, and consumer-wearable settings, learning unified representations that transfer across contexts without retraining. CardioFM achieves an F1-score of 0.86 for cardiovascular disease classification on PTB-XL, estimates the QT interval with a mean error of 20.2 ms approaching expert inter-observer variability, and measures pulse arrival time with a mean error of 22.7 ms sufficient to support non-invasive hemodynamic trending. When used as a feature extractor, CardioFM embeddings provide superior discrimination for intensive care false alarm reduction compared with ECG-FM, PaPaGei, and TimesFM, despite requiring substantially smaller representations. In contrast, generic temporal pretraining fails to encode clinically relevant waveform morphology. Demographic inference from waveform embeddings (age MAE: 10.4 years; gender AUC: 0.97; BMI MAE: 0.66 kg/m^2^) confirms that the learned representations encode fundamental biological characteristics without requiring diagnostic labels. The model maintains zero-shot reconstruction fidelity across five independent datasets spanning heterogeneous sensor hardware, sampling rates, and patient populations, with the cross-modal attention mechanism providing robustness to single-modality signal degradation. The 17.11-million-parameter encoder is compatible with edge-deployment constraints, and the model uses only signal modalities already acquired by standard bedside monitors and consumer wearables, requiring no additional sensing hardware. These findings demonstrate that a single multimodal foundation model can consolidate the fragmented landscape of cardiac biosignal analysis, providing a unified representational framework across clinical monitoring systems and wearable health technologies that may extend to broader critical illness surveillance.

## Introduction

Cardiovascular diseases account for approximately 18.6 million deaths annually, representing nearly one-third of global mortality [1]. Electrocardiography (ECG) and Photoplethysmography (PPG) serve complementary diagnostic roles: ECG captures myocardial depolarization-repolarization sequences essential for arrhythmia characterization and conduction abnormality detection, while PPG quantifies pulsatile arterial blood volume through optical absorption, providing indices of peripheral perfusion, arterial compliance, and autonomic tone [2, 3]. Although ECG and PPG signals are closely linked, arising from the same heartbeat, there is a lack of comprehensive computational models that address their interactions.

Contemporary cardiac monitoring spans heterogeneous acquisition environments. Clinical multi-lead ECG systems yield high-fidelity diagnostic recordings, whereas intensive care units increasingly deploy reduced-lead configurations alongside continuous PPG for hemodynamic surveillance [4]. Consumer wearables have extended ambulatory monitoring into community settings, generating longitudinal ECG and PPG data at unprecedented scale [5]. This proliferation of sensing modalities has outpaced analytical methodologies optimized for standardized inputs.

Traditional methods for classifying cardiac biosignals, often using convolutional or recurrent neural networks trained on carefully curated datasets with expert annotations, face three main limitations [6]. First, architectural rigidity precludes generalization across variable lead configurations; models trained on multi-lead data cannot accommodate single-lead wearable recordings without substantial modification. Second, dependence on labor-intensive annotation fails to exploit the abundance of unlabeled physiological recordings accumulating across clinical repositories [7]. Third, task-specific model proliferation, with separate networks for different modalities, devices, and clinical endpoints, impedes deployment in resource-constrained settings where the cardiovascular disease burden is rising most rapidly [8].

Foundation models, pretrained on large-scale heterogeneous corpora via self-supervised objectives, have demonstrated robust transfer learning across natural language and vision domains [9-12]. Medical applications have emerged in different medical domains [13], yet cardiac biosignal foundation models remain nascent. Three distinct paradigms have emerged. ECG-specific models such as ECG-FM [14] employ masked reconstruction and contrastive pretraining on multi-lead recordings from clinical databases, learning rich electrophysiological representations but inheriting the dimensional constraints of their training modality [15]. PPG-specific models such as PaPaGei [16] use morphology-aware self-supervised learning to encode photoplethysmographic waveform characteristics, but cannot process ECG signals or capture cross-modal physiological coupling. Domain-agnostic time-series models such as TimesFM [17] apply general-purpose forecasting objectives at a massive scale, yet lack exposure to physiological signals during pretraining. None of these approaches integrates both ECG and PPG or leverages the cross-modal physiological complementarity when both signals are acquired simultaneously.

Beyond these technical limitations, there is a compelling clinical argument for a unified foundation model. In current intensive care and perioperative settings, bedside clinicians deal with a vast amount of monitoring data from a disjointed system of specialized algorithms. Each algorithm is based on different datasets and managed by various vendors, leading to outputs that may conflict rather than work together [18, 19]. A patient in a cardiac ICU may simultaneously receive hemodynamic instability alerts, arrhythmia notifications, QT-prolongation warnings, and false-alarm suppression outputs from four independent systems, none of which shares a representational substrate with the others [18-20]. This algorithmic fragmentation contributes to alarm fatigue [19, 20], increases integration complexity, and impedes the coordinated interpretation of multimodal physiological data at the moment of clinical decision-making. A single foundation model that learns from both ECG and PPG could simultaneously support continuous drug-safety monitoring [21, 22], hemodynamic trending [23], arrhythmia detection [24], myocardial injury detection [25, 26] and alarm suppression [27, 28] from a unified pretrained representation, reducing the need for separate task-specific systems. When one type of signal deteriorates due to factors such as electrosurgical interference, motion artifacts, or reduced blood flow during vasopressor use, a bidirectional cross-modal architecture is designed to leverage the intact signal, though the extent and limits of this capability under real-world conditions, including complete modality absence, require formal evaluation. Importantly, the intensive care populations from which such a model would learn are not limited to primary cardiac disease. Repositories such as the multiple tertiary sites across the USA encompass patients with sepsis, acute respiratory failure, trauma, and post-surgical hemodynamic instability, in whom ECG and PPG signals reflect systemic autonomic tone and peripheral perfusion as much as cardiac-specific pathology [29, 30]. A model pretrained on this heterogeneous cohort may therefore encode representations relevant to critical illness monitoring beyond primary cardiac end-points, though evaluating this broader hypothesis remains a direction for future work. The potential to simultaneously reduce algorithmic fragmentation, lower deployment complexity, and extend high-fidelity monitoring to resource-limited settings where the cardiovascular burden is growing most rapidly [1] constitutes the primary clinical motivation for the work presented here.

**Figure 1:**
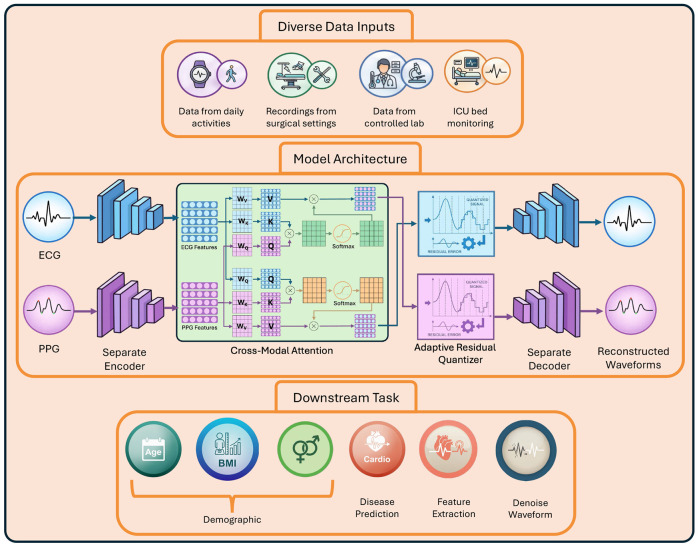
A multimodal framework integrates heterogeneous physiological data from dailylife, laboratory, surgical, and ICU settings. Modality-specific ECG and PPG encoders are fused via cross-modal attention, refined with adaptive residual quantization, and decoded for reconstruction. The learned representations enable robust clinical estimation of demographics, disease prediction, feature extraction, and waveform denoising.

We present CardioFM, shown in Figure 1, a self-supervised multimodal foundation model designed for heterogeneous ECG–PPG analysis. The architecture couples modality-specific one-dimensional convolutional encoders with a transformer-based cross-modal attention module supporting bidirectional information exchange [31, 32]. A discrete bottleneck implemented via adaptive residual vector quantization produces compact token sequences that can be efficiently stored and reused for downstream tasks; full architectural details, including the quantization mechanism and training regularization, are provided in the Methods section. The model is implemented as a 17.11-million-parameter encoder operating on ECG Lead-II and PPG inputs. Because both signals are routinely acquired by commodity bedside monitors and consumer wearables, clinical deployment requires no additional sensing hardware and the model fits within the memory budget of edge-class inference platforms.

Pretraining employs temporally aligned ECG–PPG waveforms curated from intensive care monitoring, ambulatory recordings, controlled laboratory acquisitions, and consumer wearable devices, totaling over 500,000 hours from approximately 63,000 patients. Data are partitioned at the patient level to prevent leakage between training and evaluation sets. The pretraining objective combines reconstruction loss across modalities with quantization commitment terms, yielding both continuous fused embeddings and discrete token indices exportable for downstream modeling. The standardized preprocessing pipeline, including resampling, baseline-wander correction, and powerline interference suppression, is described in full in the Methods.

Here, we show that CardioFM achieves state-of-the-art cardiovascular disease classification on the PTB-XL benchmark (F1 = 0.86), clinically meaningful QT interval estimation (MAE = 20.2 ms) and pulse arrival time measurement (MAE = 22.7 ms), and superior feature extraction for ICU false alarm reduction compared with ECG-FM, PaPaGei, and TimesFM—despite using substantially smaller representations. The model demonstrates strong demographic inference from waveform embeddings, confirming that the self-supervised representations encode fundamental biological characteristics and maintain zero-shot reconstruction fidelity across five independent datasets spanning heterogeneous sensor hardware, sampling rates, and patient populations.

## Result

### Self-supervised pretraining on multi-source ECG–PPG waveforms

The model is pretrained using a self-supervised reconstruction objective that learns representations from paired ECG–PPG waveforms without requiring diagnostic labels. Experimental data were aggregated from multiple clinical waveform repositories, including intensive care monitoring records from multiple tertiary sites across the USA, waveform archives compatible with MIMIC-IV [29] formatting conventions, PTB-XL [33], VitalDB [30], PPG-DaLiA [34], VTaC [35], and WESAD [36]. These sources offer paired ECG and PPG recordings collected in various clinical settings, including during surgery, in intensive care, during everyday activities, and in controlled laboratory stress tests. The recordings were obtained using different device setups and protocols. To ensure consistent model input, all waveforms underwent a standardized preprocessing pipeline before segment extraction. Full details of the model architecture, including the modality-specific encoders, cross-modal attention mechanism, adaptive residual vector quantization (RVQ), and the composite pretraining loss (reconstruction and commitment terms), are provided in the Methods section.

### Downstream task evaluation and clinical applicability

**Figure 2:**
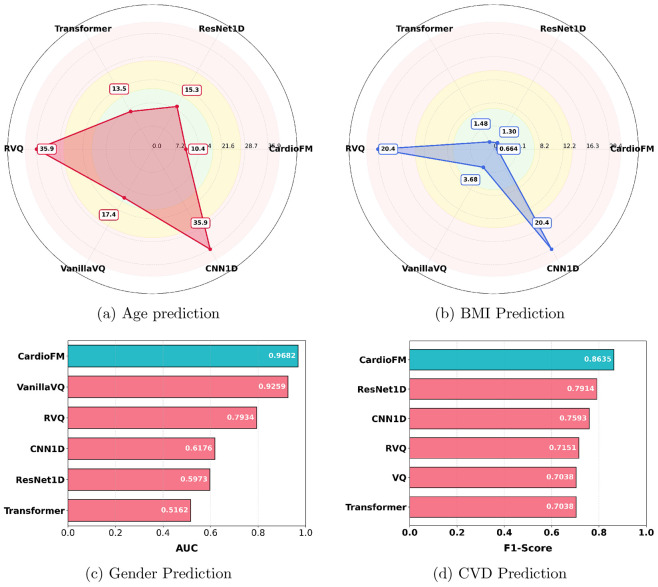
Downstream task performance across demographic inference and disease classification benchmarks. (a) Age prediction MAE (years) on PPG-DaLiA. CardioFM: 10.4; best baseline: 13.5 (Transformer). (b) BMI prediction MAE (kg/m^2^) on PPG-DaLiA. CardioFM: 0.664; best baseline: 1.30 (ResNet1D). (c) Gender classification AUC on PPG-DaLiA. CardioFM: 0.9682; best baseline: 0.9259 (VanillaVQ). (d) CVD classification F1-score on PTB-XL. CardioFM: 0.8635; best baseline: 0.7914 (ResNet1D).

**Figure 3:**
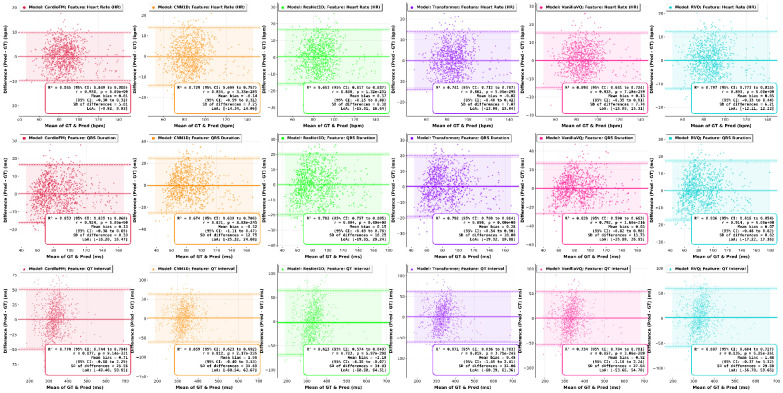
Comparison of CardioFM and baseline models using Bland–Altman analysis for ECG-derived electrophysiological interval estimation.

**Figure 4:**
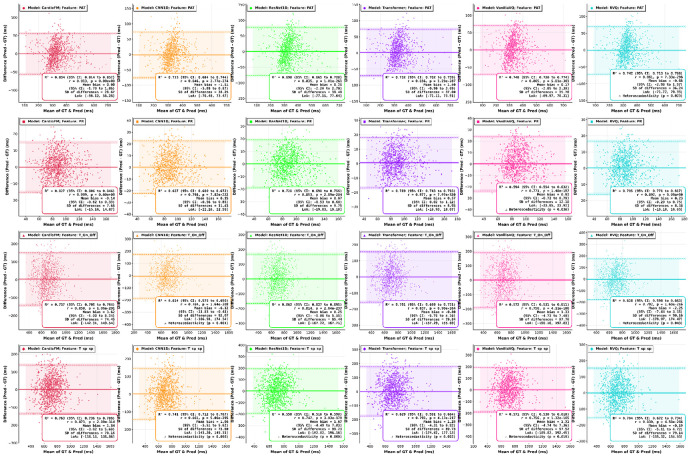
Comparison between CardioFM and baseline models of Bland-Altman agreement analysis for PPG-derived hemodynamic parameter extraction.

CardioFM was designed for robust generalization across heterogeneous sensing devices and clinical contexts, facilitating deployment in diverse healthcare environments. To assess this capability, the pretrained model was evaluated on downstream tasks spanning four domains: demographic attribute inference (age, gender, body mass index), cardiovascular disease classification (PTB-XL benchmark) (Figure 2), physiological feature extraction (heart rate, QT interval, and pulse arrival time (PAT) from paired ECG-PPG recordings) (Figure 3 and 4), and waveform denoising (5). These tasks collectively probe the model’s capacity to encode meaningful morphological and temporal patterns underlying diagnostic decision-making and continuous physiological monitoring. Architecturally, denoising employed convolutional layers appended to the encoder for sequence-to-sequence regression, whereas classification and scalar regression tasks used a single fully connected layer. Evaluation datasets encompassed varied acquisition protocols, device configurations, and patient populations to comprehensively assess clinical transferability.

### Comparative evaluation against baseline architectures

We assessed CardioFM’s adaptability across clinically relevant tasks by systematically comparing it with established deep learning architectures trained from scratch, including one-dimensional convolutional networks (CNN1d), residual networks (ResNet1d), Transformer-based models, and quantization methods (VQ, RVQ). This experimental design addresses a fundamental constraint in clinical biosignal analysis: conventional approaches assume fixed input configurations and predefined prediction targets, precluding direct transfer across clinical scenarios without substantial architectural modification. By comparing fine-tuned CardioFM against task-specific baselines, we examined whether foundation model pretraining confers practical advantages across varied clinical contexts and task requirements. CardioFM’s unified representational framework circumvents these limitations, readily adapting to varied clinical contexts and task requirements. These findings demonstrate the potential of pretrained cardiac foundation models as flexible analytical tools supporting diverse biosignal interpretation needs across healthcare settings.

### Demographic attribute inference from physiological waveforms

To assess whether the pretrained representations encode fundamental biological characteristics, we evaluated CardioFM on demographic recognition tasks using the PPG-DaLiA dataset. Data were partitioned at the subject level, allocating 80% for training, 10% for validation, and 10% for testing. Three attributes were examined independently: age and body mass index (BMI), both quantified by mean absolute error (MAE), where lower values indicate superior performance, and gender, assessed by area under the receiver operating characteristic curve (AUC). CardioFM demonstrated consistent improvements over all baseline architectures trained from scratch across each demographic attribute. For age estimation, CardioFM achieved an MAE of 10.42 years, outperforming Transformer (13.51), ResNet1D (15.31), VanillaVQ (17.44), CNN1D (35.90), and RVQ (35.92). The advantage was particularly pronounced for BMI prediction, where CardioFM attained an MAE of 0.66 compared with substantially higher errors from ResNet1D (1.30), Transformer (1.48), VanillaVQ (3.86), and the poorest-performing models, CNN1D (20.36) and RVQ (20.41). For gender classification, CardioFM achieved an AUC of 0.9682, exceeding VanillaVQ (0.926), RVQ (0.793), CNN1D (0.618), ResNet1D (0.597), and Transformer (0.516). The findings shown in Figure 2a, 2b, and 2c indicate that self-supervised pretraining helps the model identify essential patterns in age, body composition, and gender. The findings indicate that self-supervised pretraining effectively captures important biological characteristics from raw waveforms. These characteristics include age-related changes in conduction, differences in vascular compliance based on body composition, and electrophysiological variations linked to sex. This confirms that the learned representations have a biological basis before being applied to urgent clinical tasks.

### Cardiovascular disease prediction

We evaluated CardioFM on the PTB-XL dataset, a widely adopted benchmark for electrocardiogram interpretation comprising a multi-label classification framework. To ensure unbiased performance estimation, data were partitioned at the subject level, with 80% allocated for training, 10% for validation, and 10% reserved for held-out testing. CardioFM achieved the highest F1-score of 0.86, substantially outperforming (Figure 2d) all baseline architectures trained from scratch on the same data. Among the comparison models, ResNet1D achieved 0.79, followed by CNN1D at 0.76, while quantization-based approaches performed worse, with RVQ at 0.72 and VanillaVQ at 0.70. The Transformer architecture similarly obtained an F1-score of 0.70. Across most evaluation scenarios, CardioFM consistently outperformed conventional supervised learning methods, with the full model configuration achieving the highest F1-score among all tested variants. These findings indicate that representations learned through self-supervised pretraining on heterogeneous waveform repositories transfer effectively to diagnostic classification tasks, even when fine-tuned on moderately sized clinical datasets.

### Physiologically Interpretable Feature Extraction from ECG and PPG

Analysis of biomedical signals, particularly ECG and PPG, has advanced considerably over the years. Manual extraction of commonly used domain-specific features is still a popular approach. However, achieving robustness and generalizability across diverse data collection protocols and devices remains a significant challenge. Limited efforts have been made to develop a unified toolbox for extracting useful features from such heterogeneous settings, whether through traditional manual feature engineering or deep neural networks. We demonstrate that the representations learned by our pretrained models serve as effective embeddings, accommodating these variations and enhancing performance across different settings.

#### ECG feature extraction:

To quantify how faithfully learned representations preserve cardiac waveform characteristics, we evaluated CardioFM on regression tasks targeting three electrophysiologically significant intervals: QT interval (ventricular depolarization-repolarization duration, a marker of arrhythmogenic risk), QRS complex duration (ventricular conduction integrity, predictive of heart failure outcomes), and instantaneous heart rate derived from R-R periodicity (reflecting autonomic tone and cardiac output). Performance was assessed using the coefficient of determination (R^2^), MAE, and RMSE to comprehensively characterize explained variance and prediction accuracy.

CardioFM achieved superior performance across all three regression targets compared to baseline architectures initialized randomly that shown in Table 1 . For QT interval estimation, CardioFM yielded an R^2^ of 0.770, corresponding to an MAE of 20.22 ms and RMSE of 25.56 ms. This represents a relative reduction in MAE of 26.3% compared with ResNet1D (R^2^ = 0.613, MAE = 27.42 ms, RMSE = 34.08 ms) and 8.5% compared with VanillaVQ (R^2^ = 0.734, MAE = 22.11 ms, RMSE = 27.64 ms), the second-ranked model. The magnitude of these errors falls within the range of inter-observer variability reported for manual QT annotation, suggesting clinical applicability shown in Figure 3. Heart rate regression exhibited the highest explained variance among the three tasks, with CardioFM attaining R^2^ = 0.865, MAE = 4.01 bpm, and RMSE = 5.01 bpm. Relative to ResNet1D (R^2^ = 0.653, MAE = 6.74 bpm), this constitutes a 40.6% reduction in absolute error, while the improvement over RVQ (R^2^ = 0.797, MAE = 4.97 bpm) was 19.4%. The superior performance in heart rate estimation likely reflects the quasi-periodic structure of cardiac signals, which self-supervised reconstruction objectives are well-positioned to capture through temporal coherence constraints. QRS duration prediction demonstrated similarly robust results, with CardioFM achieving R^2^ = 0.853, MAE = 6.72 ms, and RMSE = 8.33 ms. Notably, quantization-based architectures showed variable performance on this task. RVQ achieved results close to CardioFM (R^2^ = 0.836, MAE = 7.03 ms). In contrast, VanillaVQ and CNN1D demonstrated lower accuracy, with MAE values of 10.78 ms and 10.11 ms, corresponding to 60.4% and 50.5% higher error than CardioFM, respectively.

#### PPG feature extraction:

Beyond electrocardiographic parameters, we assessed CardioFM’s capacity to extract hemodynamically relevant features derived from photoplethysmographic waveform morphology (Figure 4). Four regression targets were examined: PAT, pulse rate, pulse duration (Ton off ms, defined as the interval from pulse onset to offset), and inter-systolic interval (Tsp sp ms, quantifying the temporal separation between consecutive systolic peaks). These parameters encode complementary information regarding cardiovascular dynamics. Performance was quantified using R^2^, MAE, and RMSE to comprehensively characterize regression accuracy.

CardioFM demonstrated consistently superior performance across pulse wave parameters, as shown in Table 2.For PAT estimation, the model achieved R^2^ = 0.834, MAE = 22.74 ms, and RMSE = 28.66 ms, representing 27.8% and 20.7% reductions in MAE compared with ResNet1D (R^2^ = 0.698, MAE = 31.48 ms) and VanillaVQ (R^2^ = 0.748, MAE = 28.67 ms), respectively. Given that PAT-derived blood pressure algorithms exhibit sensitivities of 0.5–1.0 mmHg per millisecond, these improvements translate into clinically meaningful gains for downstream hemodynamic inference. Pulse rate regression yielded R^2^ = 0.827 and MAE = 6.09 bpm, reflecting 22.3% improvement over ResNet1D (MAE = 7.84 bpm). Notably, VanillaVQ and CNN1D exhibited degraded performance (MAE = 9.67 and 9.15 bpm, respectively), suggesting standard quantization schemes without residual refinement inadequately preserve temporal resolution for accurate rate estimation. For morphology parameters requiring precise fiducial localization, CardioFM maintained performance advantages despite increased complexity. Pulse duration estimation achieved R^2^ = 0.737 and MAE = 59.48 ms, outperforming the Transformer model (MAE = 63.77 ms) by 6.7% and ResNet1D (MAE = 67.31 ms) by 11.6%. In contrast, VanillaVQ showed marked performance degradation (R^2^ = 0.572, MAE = 79.02 ms), corresponding to a 32.9% increase in error, likely due to information loss during discretization of subtle waveform features that define pulse boundaries. Inter-systolic interval regression demonstrated similar patterns: CardioFM attained R^2^ = 0.763 and MAE = 55.44 ms, exceeding ResNet1D (R^2^ = 0.558, MAE = 79.42 ms) by 30.2%. While CNN1D performed competitively (R^2^ = 0.741, MAE = 58.98 ms), suggesting convolutional architectures effectively capture periodic structures, CardioFM maintained a 6.0% advantage.

The performance across both ECG-derived intervals and PPG-derived pulse wave features indicates that the cross-modal attention mechanism effectively aligns temporal representations between modalities, enabling the pretrained encoder to capture the physiological coupling between electrical cardiac activity and peripheral hemodynamic manifestations. These findings suggest that CardioFM learns a unified cardiovascular representation that is amenable to diverse downstream feature-extraction tasks spanning the cardiac conduction-to-peripheral circulation pathway.

### Zero-shot cross-dataset waveform denoising fidelity

**Figure 5:**
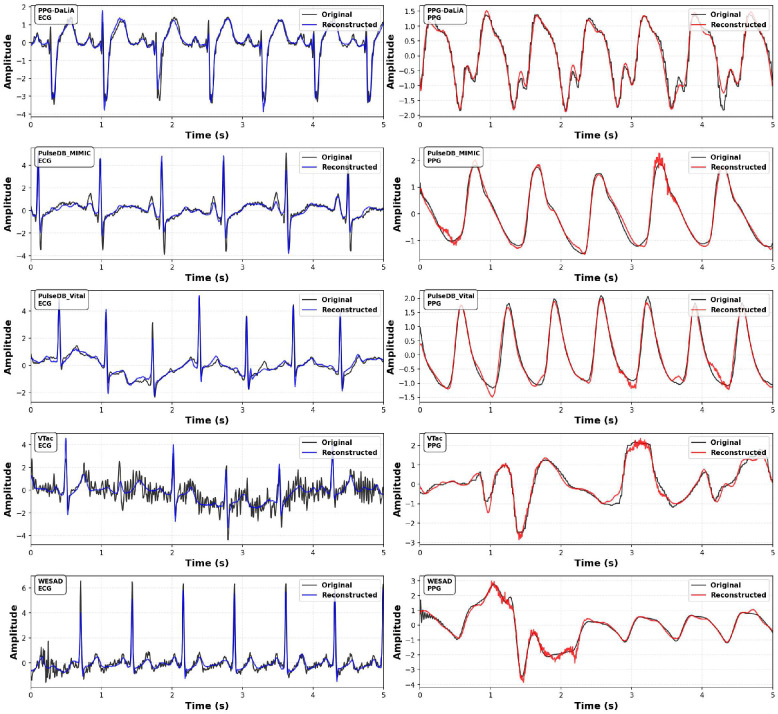
Qualitative denoising of ECG and PPG signals across heterogeneous cardiovascular datasets.

To assess representational capacity and transferability, we evaluated zero-shot waveform denoising fidelity across five independent datasets spanning heterogeneous acquisition contexts: PPG-DaLiA (ambulatory activities), PulseDB MIMIC (intensive care monitoring), PulseDB VitalDB (intraoperative recordings), VTaC (ICU arrhythmia events), and WE-SAD (laboratory stress protocols). The pretrained encoder was applied without fine-tuning or parameter adjustment, enabling assessment of generalization to unseen data, as shown in Figure 5.

CardioFM effectively preserved key morphological features in ECG and PPG reconstructions across diverse datasets without specific adaptation. In ECG reconstruction, it accurately reproduced the QRS complex, including the P-, and T-wave components, maintaining fidelity despite significant baseline wander and motion artifacts. Notably, noise in the PulseDB Vital and VTaC datasets did not affect adjacent stable segments, and the model performed well with varying R-peak amplitudes and QRS shapes. For PPG, CardioFM successfully captured critical blood flow dynamics, including the systolic upstroke, peak, and diastolic decay phases, as well as the dicrotic notch, which is important for vascular health. The reconstructions from WESAD and VTaC showed strong fidelity in capturing beat-to-beat variability, including subtle amplitude changes from respiratory sinus arrhythmia. This ability to preserve fine morphological details under zero-shot conditions indicates the model’s learned representations generalize well beyond the characteristics of the pretraining corpus.

CardioFM demonstrates consistent zero-shot reconstruction across varied datasets from different sensor hardware (clinical-grade ICU monitors versus consumer wearable devices), sampling frequencies (64 Hz to 500 Hz), patient populations (surgical patients, critically ill individuals, healthy ambulatory subjects), and physiological states (rest, physical activity, acute stress). This indicates that its self-supervised pretraining creates domain-invariant representations.

### CardioFM as a Feature Extractor

**Figure 6:**
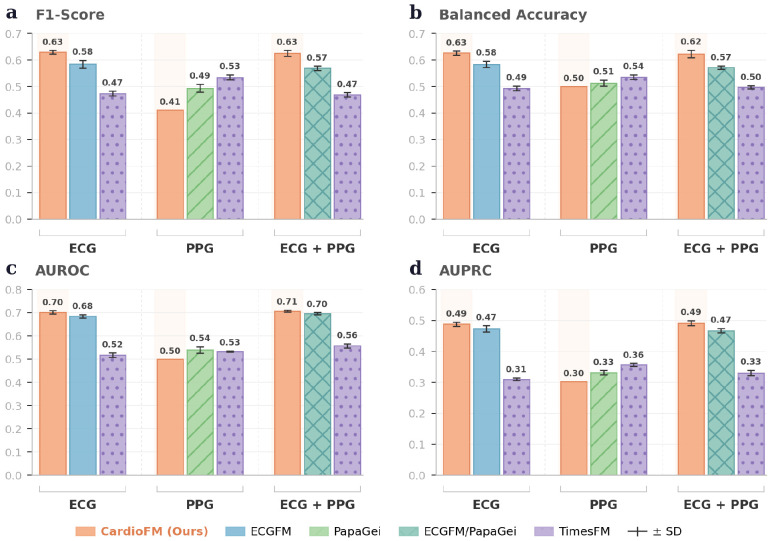
Comparative performance of CardioFM across unimodal and multimodal inputs. Performance of CardioFM (ours), ECGFM, PapaGei, ECGFM/PapaGei, and TimesFM across three input configurations (ECG, PPG, and combined ECG+PPG) evaluated using four metrics: (a) F1-score, (b) balanced accuracy, (c) AUROC, and (d) AUPRC. CardioFM performed the best across modalities, especially in the multimodal ECG+PPG setting. In unimodal ECG evaluation, CardioFM outperformed domain-specific and general time-series foundation models across all metrics. In unimodal PPG, performance differences narrowed, although CardioFM remained competitive. Multimodal integration (ECG+PPG) yielded the highest discrimination overall, highlighting the benefit of joint representation learning across complementary cardiac signals. These results demonstrate the robustness and generalizability of CardioFM across sensing modalities and underscore the performance advantage of a purpose-built multimodal cardiac foundation model over modality-specific and domain-agnostic baselines.

A major challenge in using foundation models in clinical practice is determining if their representations can be effective without specific fine-tuning or transfer learning. We investigated whether frozen embeddings from CardioFM could serve as useful off-the-shelf features for distinguishing ICU false alarms on the VTaC dataset. We compared these embeddings with ECG-FM [14], PaPaGei [16], and the domain-agnostic TimesFM [17] under the same evaluation conditions, using an XGBoost classifier for analysis (Figure 6).

CardioFM ECG-only embeddings achieved an F1-score of 0.63 ± 0.01 and area under the receiver operating characteristic curve (AUROC) of 0.7 ± 0.01, surpassing ECG-FM (F1: 0.584 ± 0.015; AUROC: 0.685 ± 0.007) despite using less than one-third of ECG-FM’s embedding dimensionality (256 vs. 768). The CardioFM multimodal configuration (512-dimensional, ECG+PPG) achieved the highest AUROC (0.71 ± 0.01) and area under the precision–recall curve (AUPRC) (0.49 ± 0.01) of all evaluated systems, outperforming the concatenation of two independent modality-specific encoders (ECG-FM + PaPaGei; F1-score: 0.57 ± 0.01; AUROC: 0.7 ± 0.004; embedding dimension: 1,280). It demonstrates that joint multimodal pretraining produces more transferable representations than post-hoc feature fusion. TimesFM embeddings, derived from a domain-agnostic forecasting objective, performed near-chance across all modalities (ECG-only AUROC: 0.52), confirming that generic temporal pretraining objectives do not encode morphological information in physiological waveforms.

Using only PPG resulted in consistently poor performance across all encoders. This is due to PPG signals lacking the direct electrophysiological markers that are essential for identifying ventricular tachycardia. This identification primarily depends on the characteristics of the QRS complex and features of atrioventricular dissociation, which PPG cannot detect.

### ECG-PPG Embedding Quality Across CardioFM Fusion Architectures

**Figure 7:**
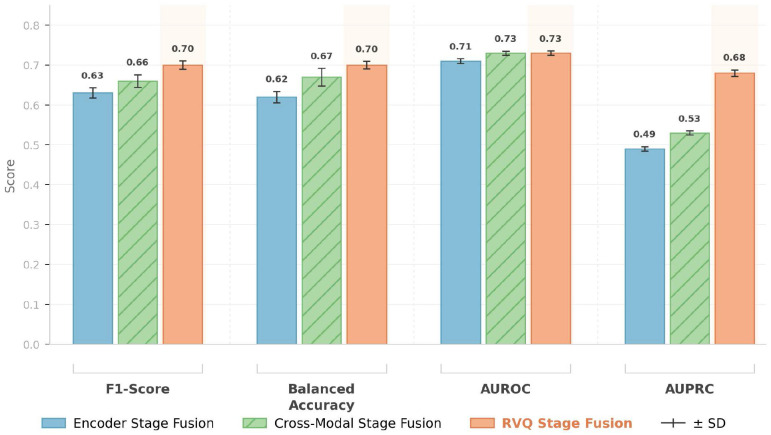
Representation quality across CardioFM stages shows progressive gains from encoder to cross-modal to RVQ fusion. RVQ achieves best performance across all metrics, especially AUPRC (0.68), indicating improved precision in imbalanced settings. Error bars denote ±SD; improvements reflect both deeper processing and fusion-specific contributions.

To characterize how representational quality evolves through CardioFM’s processing stages, we compared embeddings extracted from three successive points within the trained model under a linear probing protocol: encoder-level fusion (Encoder Fused), cross-modal attention-based fusion (Cross-Modal Fused), and residual vector quantization-based fusion (RVQ Fused) (Figure 7). Because these embeddings are extracted from progressively deeper layers of the same trained network, performance differences may reflect both the specific architectural mechanism at each stage and the cumulative effect of additional nonlinear transformations. We therefore interpret these results as a representation quality analysis across processing stages rather than a strict component ablation. RVQ Fused embeddings consistently achieved the highest performance across all metrics, attaining an F1-score of 0.70, balanced accuracy of 0.70, AUROC of 0.73, and AUPRC of 0.68, outperforming both other layers by non-trivial margins. Encoder Fused embeddings yielded the lowest discriminative capacity, particularly on AUPRC (0.49), suggesting that naive concatenation of unimodal representations introduces redundancy and fails to capture the complementary structure inherent in jointly acquired ECG-PPG signals. Cross-Modal Fused embeddings showed a moderate improvement, achieving an AUROC of 0.73, which is the same as that of RVQ Fused. However, its AUPRC score was only 0.53. This indicates that although attention-based fusion can effectively capture overall relationships, it struggles with precision when class sizes are imbalanced, a frequent issue in cardiac phenotyping tasks. The progressive improvement across processing stages is consistent with the interpretation that each architectural component contributes distinct representational value, though we note that depth and mechanism are inherently confounded when extracting embeddings from a single trained model. Two observations support a mechanism-specific interpretation. First, the largest performance gap occurred on AUPRC (0.49 → 0.53 → 0.68), a metric sensitive to precision in imbalanced settings, where discrete quantization’s event-level tokenization would be expected to help most. Second, it is important to note that baseline models trained independently with static RVQ, without using cross-modal attention or adaptive codebooks, performed significantly worse on all downstream tasks. For example, the F1 score was 0.72, while CardioFM achieved 0.86 on the PTB-XL dataset. Furthermore, the mean absolute error (MAE) for age prediction was 35.9 years for the baseline models, compared to just 10.4 years for CardioFM, even though both models included a quantization layer at a similar depth in the network. This indicates that it is not simply the presence of a deeper quantization layer that drives performance, but rather the specific combination of cross-modal attention with adaptive residual vector quantization that produces the representational advantage. A definitive disentanglement of depth and mechanism effects would require training separate models with each component removed independently, which we identify as an important direction for future work.

## Discussion

CardioFM demonstrates generalization across diverse clinical scenarios, devices, and input configurations. By integrating heterogeneous ECG–PPG data through cross-modal attention and adaptive residual vector quantization, the model addresses the fragmentation inherent in conventional approaches that require modality-specific architectures and task-specific optimization [9, 13]. The pretraining cohort is not limited to patients with primary cardiac pathology. The training data for CardioFM were obtained from multiple tertiary sites across the USA, comprising patients admitted to the ICU. These repositories include patients with conditions such as sepsis, acute respiratory failure, trauma, and post-surgical hemodynamic instability. In these cases, ECG and PPG signals primarily reflect systemic autonomic tone, intravascular volume status, and peripheral perfusion rather than isolated cardiac disease [30]. This cohort heterogeneity means that the self-supervised objective was exposed to cardiovascular waveform signatures spanning a broad range of critical illness phenotypes, not only primary cardiac events. Although the downstream evaluations presented here focus on cardiac-specific endpoints (disease classification, interval estimation, false alarm reduction), the diversity of the pretraining corpus provides a principled basis for the hypothesis that CardioFM’s representations also encode hemodynamic and autonomic patterns relevant to non-cardiac critical illness. Validating this hypothesis through downstream evaluation on endpoints such as sepsis onset, hemodynamic decompensation, and vasopressor responsiveness is an important next step, and we discuss specific opportunities below.

Extensive evaluations confirm consistent performance advantages over conventional deep learning architectures. On PTB-XL, CardioFM achieved an F1-score of 0.86 versus 0.79 for ResNet1D, likely reflecting richer temporal representations learned through cross-modal fusion. Analysis of representation quality across CardioFM’s processing stages (Figure 7) reveals progressive improvement from the encoder level through cross-modal attention to RVQ-based fusion across all evaluation metrics. Because these representations are extracted from successive layers of the same trained model, the improvement reflects a combination of cumulative processing depth and mechanism-specific contributions. However, the comparison with independently trained static quantization baselines shows that while these baselines have quantization layers of similar depth, they perform significantly worse (e.g., F1 = 0.72 compared to 0.86 on PTB-XL). This indicates that the adaptive RVQ mechanism offers benefits beyond depth alone. A plausible physiological explanation for this advantage is that cardiac physiology is inherently organized around discrete, cyclical events. These events include individual heartbeats, systolic upstrokes, diastolic decays, and QRS complexes, each constituting a quasi-discrete morphological unit. The discrete tokenization imposed by residual vector quantization may naturally align with this event-level structure, effectively learning a vocabulary of recurring cardiac morphological states (e.g., normal sinus beats, ectopic complexes, varying pulse amplitudes) that downstream classifiers can exploit directly. Continuous embeddings, by contrast, distribute morphological information diffusely across the latent space, requiring additional representational work to recover event-level features. This physiological interpretation is one of several possible explanations; the information-theoretic regularization properties of discrete bottlenecks and the monotonic approximation refinement inherent to residual quantization may also contribute to the observed advantage. Investigating these factors through targeted ablations, such as comparing adaptive and fixed codebooks at the same quantization depth, would be an important direction for future research.

The demographic inference results from PPG-DaLiA indicate a mean absolute error (MAE) of 10.4 years for age, 0.66 kg/m^2^ for BMI, and an area under the curve (AUC) of 0.97 for gender classification. These results confirm that self-supervised pretraining on raw waveforms, without any diagnostic labels, effectively captures important biological characteristics. This includes changes in conduction related to age, age-dependent vascular compliance, and sex-related electrophysiological differences. These attributes are often available in electronic health records and are not clinical targets by themselves. Accurately extracting them from waveform embeddings provides the biological foundation for developing downstream acute clinical tasks with confidence.

These technical gains translate into clinically meaningful capabilities. The QT interval estimation error (MAE = 20.2 ms) approaches the inter-observer variability reported for expert manual annotation [37], a threshold that has long limited the reliability of automated QT assessment. The pulse arrival time accuracy (MAE = 22.7 ms) is similarly significant. Given that PAT-derived blood pressure algorithms exhibit subject-specific sensitivities on the order of 0.5–3.0 mmHg per ms depending on the arterial path, patient demographics, and calibration method [38], improvements in PAT estimation accuracy translate into clinically meaningful gains for downstream hemodynamic inference. This level of precision supports non-invasive hemodynamic trending as a potential surrogate for invasive arterial catheterization, a procedure that carries risks of vascular injury, thrombosis, and infection. Recent reviews from the European Society of Hypertension have underscored the urgent need for validated cuffless blood pressure technologies [39]. Zero-shot reconstruction fidelity across five independent datasets from ICU monitors, surgical recordings, and consumer wearables indicates domain-invariant representations that generalize beyond pretraining characteristics, similar to the cross-site transfer performance reported by recent foundation models in sleep medicine and ECG analysis [40, 41]. The performance across both ECG-derived intervals and PPG-derived pulse wave features indicates that the cross-modal attention mechanism effectively aligns temporal representations between modalities, enabling the pretrained encoder to capture the physiological coupling between myocardial electrical activity and peripheral hemodynamic manifestations.

Beyond these quantitative benchmarks, the translational significance of CardioFM lies in its capacity to consolidate the fragmented monitoring ecosystem that currently burdens bedside clinicians. In a typical ICU, a patient may simultaneously receive hemodynamic instability alerts, arrhythmia notifications, QT-prolongation warnings, and false-alarm suppression outputs from independent systems trained on different datasets and maintained by different vendors, resulting in outputs that may conflict rather than align [18, 19]. This algorithmic fragmentation contributes directly to alarm fatigue and 88.8% of the 12,671 annotated arrhythmia alarms were false positives [42]. This desensitization has been linked to delayed responses and preventable patient deaths [28]. A single foundation model deriving multiple clinical assessments from one pretrained representation addresses this fragmentation at its source: the clinician interacts with one coherent interpretive framework that shares information across tasks. Crucially, both ECG and PPG are routinely and continuously acquired at monitored bedsides. The American Heart Association recommends continuous ECG monitoring for all ICU patients [43], and pulse oximetry, which captures the PPG waveform during SpO2 measurement, is universally used in critical care [44]. Therefore, CardioFM requires no additional sensing hardware and leverages data streams that are already being collected but remain diagnostically underutilized.

Several high-impact clinical applications illustrate the potential of this unified approach. First, CardioFM’s accurate QT interval estimation enables continuous, automated monitoring for drug-induced QT prolongation, a concern affecting 27.9% of critically ill patients receiving antipsychotics, macrolides, or fluoroquinolones [45]. This risk is difficult to detect reliably with intermittent 12-lead ECG workflows. Recent work has demonstrated the feasibility of AI-enabled QT assessment from simplified ECG inputs [46], and CardioFM extends this paradigm by integrating concurrent PPG-derived hemodynamic features that may further contextualize arrhythmogenic risk. Second, the model’s pulse arrival time accuracy supports non-invasive hemodynamic trending for patients who would benefit from continuous blood pressure monitoring but do not meet criteria for invasive access [47]. Third, the multimodal embeddings jointly capturing arrhythmia morphology and autonomic dynamics provide a rich feature space for early detection of clinical deterioration and cardiac arrest, where deep learning approaches using continuous waveforms have demonstrated AUROCs of 0.85–0.94 with substantially fewer false alarms than conventional early warning scores [48-50]. Fourth, continuous ECG and PPG feature extraction could enable real-time screening for perioperative myocardial injury (MINS), a condition affecting approximately 18% of surgical patients. More than 90% of cases are asymptomatic and therefore missed without systematic troponin screening, yet targeted anticoagulation has been shown to improve outcomes in this population [25, 26, 51]. Fifth, the composition of the pretraining cohort supports a broader clinical hypothesis. The self-supervised model was trained on waveforms from patients with various systemic illnesses, including sepsis and multi-organ dysfunction. As a result, the learned representations may capture autonomic and hemodynamic signatures that extend beyond primary cardiac disease. Emerging evidence indicates that waveform-derived features can predict sepsis onset 4–12 hours before clinical recognition [52], and real-world deployments of AI-based sepsis prediction systems have demonstrated 17–19% reductions in relative mortality [53, 54]. Although CardioFM has not yet been tested for predicting sepsis, its combined ECG and PPG representations capture both heart activity and blood flow dynamics. This makes it a valuable tool for identifying the autonomic dysregulation and microcirculatory impairment that are typical in early sepsis. Evaluating the model’s effectiveness in non-cardiac critical illness endpoints is an important step in demonstrating its usefulness beyond the cardiovascular applications already established.

To illustrate the practical utility, consider a patient admitted to the medical ICU on intravenous azithromycin for pneumonia who also receives ondansetron for nausea, resulting in two concurrent QT-prolonging exposures. Under current workflows, QT monitoring depends on intermittent ECGs ordered on clinical suspicion. CardioFM continuously analyzes the Lead II ECG from the bedside monitor. It detected a gradual increase in QTc from 440 ms toward the 500 ms safety threshold, prompting a timely review of electrolytes, potassium replacement, and a change in antibiotics. These interventions could be delayed with intermittent monitoring workflows. This scenario illustrates a broader shift in healthcare from disconnected, task-specific monitoring algorithms to a single, flexible framework that can adapt to emerging clinical questions. A prospective investigation will be needed to determine whether the concurrent availability of PPG-derived hemodynamic features further enriches such clinical risk stratification beyond what ECG alone provides.

The feature extraction experiments on the VTaC false alarm dataset provide a direct comparison against three established foundation models representing distinct pretraining paradigms. ECG-FM [14], an ECG-specific model built on the wav2vec 2.0 framework, was pretrained on approximately 1.4 million segments from public repositories, yielding 768-dimensional embeddings from a 90.9-million-parameter encoder. PaPaGei [16], a PPG-specific model employing morphology-aware contrastive learning, was trained on approximately 57,641 hours of fingertip PPG, producing 512-dimensional embeddings from a compact 5.7-million-parameter architecture. TimesFM [17], a domain-agnostic 200-million-parameter decoder-only transformer, was pretrained on approximately 100 billion time-points from non-physiological sources (web traffic, synthetic, electricity and road traffic), producing 1,280-dimensional embeddings with no exposure to physiological signals.

CardioFM’s ECG-only embeddings (F1 = 0.63, AUROC = 0.70) surpassed ECG-FM (F1 = 0.584, AUROC = 0.685) despite using less than one-third the embedding dimensionality (256 vs 768) and approximately one-fifth the parameter count (~17 million vs ~90.9 million). The multimodal configuration (512-dimensional, ECG+PPG) achieved the highest AUROC (0.71) and AUPRC (0.49) among all systems, outperforming the post-hoc concatenation of ECG-FM and PaPaGei embeddings (1,280-dimensional; AUROC = 0.70, AUPRC = 0.47). This final comparison is particularly insightful. Simply combining features from two independently pretrained, domain-specific models, each representing the best available unimodal encoders for their respective modalities, does not perform as well as a jointly pretrained model with less than half the combined embedding dimensionality. This finding indicates that cross-modal pretraining captures complementary physiological structures that cannot be recovered through post-hoc fusion. TimesFM’s near-chance performance across all modalities (ECG-only AUROC = 0.52) further confirms that generic temporal pretraining, regardless of scale, does not encode the morphological features of physiological waveforms essential for clinical discrimination. Together, these comparisons suggest that CardioFM’s performance advantage is driven primarily by its multimodal architecture and cross-modal attention mechanism, rather than by raw pretraining data volume. We emphasize that a conclusive attribution requires a controlled ablation study. Specifically, this means using a CardioFM variant pretrained solely on ECG data, matched in size to the corpus we are studying, and employing the same encoder architecture without cross-modal attention. This approach would help us isolate the impact of multimodal pretraining from other factors, such as variations in data volume and encoder design choices. This ablation represents an important analysis for future work. CardioFM’s pretrained encoder remains unchanged during adaptation to downstream tasks, allowing only lightweight classification heads to be fine-tuned. New clinical endpoints can therefore be added with minimal labeled data, an important practical consideration for hospital systems that cannot afford the costs of data curation, computation, and regulatory compliance for training custom models for each monitoring goal.

Equally important is the model’s robustness to real-world signal degradation. ECG is frequently corrupted by electrosurgical interference, electrode displacement, and patient movement, while PPG quality deteriorates during vasoconstriction, hypothermia, and vasopressor use [55, 56]. CardioFM’s bidirectional cross-modal attention architecture addresses this challenge by allowing each modality’s representation to draw on the other when its own signal is noisy or corrupted, effectively upweighting the more reliable modality in the fused representation. This property is supported by the zero-shot denoising fidelity observed across five heterogeneous datasets (Figure 5), where the model maintained reconstruction accuracy despite substantial baseline wander, motion artifacts, and amplitude variation. However, it is important to distinguish between signal degradation and complete signal absence. The current architecture requires both ECG and PPG inputs during inference. If one of these signals is partially corrupted, such as when the PPG signal is weakened by norepinephrine-induced peripheral vasoconstriction, the cross-modal attention mechanism can adjust by relying more on the intact ECG signal. In clinical scenarios involving prolonged, complete signal dropout, such as a non-invasive blood pressure cuff inflating on the same arm as the pulse oximeter and flattening the PPG waveform for 30- 45 seconds, the model’s behavior has not been systematically characterized. The architecture lacks a specific mechanism to handle null inputs, and there has been no formal evaluation under conditions where all modalities are absent. To facilitate clinical deployment, it is important to develop a validated unimodal fallback inference mode. This could be achieved using learned null tokens or systematic modality dropout during pretraining.

Several additional limitations should be acknowledged. First, model pretraining was performed exclusively on Lead II ECG signals; extending to multi-lead ECG configurations will require architectural adaptation and further validation. Second, the current architecture relies on temporally aligned ECG–PPG pairs during both pretraining and inference. Although the cross-modal attention mechanism can preferentially weight one modality when the other is degraded, the model has not been validated under complete, prolonged modality absence, as discussed above; developing robust unimodal fallback inference modes is essential before clinical deployment. Third, the interpretability of learned representations remains limited. To partially address this, we stratified performance across fusion stages and data modalities and conducted zero-shot evaluations on unseen datasets. However, further work is needed to achieve case-level explainability that meets clinical expectations. Fourth, the evaluation used retrospective benchmarks, and prospective validation under real-world ICU conditions has not yet been conducted. This includes situations with simultaneous electrosurgical interference, hemodynamic instability, and PPG degradation caused by vasopressors. Fifth, the fusion architecture analysis (Figure 7) compares embeddings extracted from different processing stages of the same trained model, where depth and mechanism effects are inherently confounded. Comparing independently trained static quantization baselines provides indirect evidence for specific contributions of mechanisms. However, to achieve a definitive understanding, it would be necessary to train separate models with each architectural component removed. We consider this analysis important for future research. Finally, computational requirements, though modest relative to large language models, may still limit accessibility in resource-constrained clinical environments where the cardiovascular burden is growing most rapidly. The pretraining corpus primarily comes from multiple tertiary sites at academic medical centers in the United States. As a result, the racial, ethnic, and socioeconomic diversity of these populations may not reflect the range of settings in which CardioFM could be used. Given known disparities in PPG signal quality across skin pigmentation levels [55] and the demographic dependence of cardiovascular waveform morphology, validation across diverse patient populations is necessary before generalizability claims can be fully supported.

Future directions include extending to additional physiological modalities, developing unimodal fallback inference modes for scenarios with missing signals, integrating with large language models for enhanced interpretability, and developing computationally efficient training strategies suitable for deployment across both resource-rich and resource-limited healthcare environments. A particularly promising direction is the fusion of high-frequency waveform data with low-frequency electronic health record (EHR) data, including laboratory values, medication administrations, and clinical documentation. CardioFM’s adaptive residual vector quantization produces compact, discrete token sequences for each 10-second waveform segment, and these quantized tokens can be treated analogously to text tokens in a sequence model. This architectural property makes CardioFM a natural foundational building block for future multimodal systems that interleave high-frequency waveform tokens with low-frequency EHR-derived tokens. To realize this vision, we must tackle several technical challenges. These include aligning waveform tokens, which arrive every 10 seconds, with EHR events that may come hours or days later. We also need to ensure that the vocabulary of learned waveform codebooks matches that of clinical text embeddings. Additionally, we must develop training methods to manage the interleaving of different token types. These issues have not been fully addressed in the current multimodal clinical AI literature. As wearable technologies continue to extend cardiovascular monitoring into ambulatory and community settings, foundation models like CardioFM may offer opportunities for non-invasive, continuous health monitoring beyond the ICU. Future efforts should explore how combining waveform-based models with structured clinical data and imaging can further enhance their clinical utility.

## Methods

### Dataset and preprocessing

A preprocessing pipeline is implemented for multimodal ECG-PPG physiological signals to produce standardized inputs for self-supervised learning. The pipeline addresses sampling rate differences, artifacts, and quality control across multiple datasets. Both modalities, ECG and PPG, are required for the model training. All signals were resampled to $fs_{\text{target}}=250\;\text{Hz}$. For downsampling $fs_{\text{original}}>fs_{\text{target}}$, an anti-aliasing filter was applied prior to resampling to prevent spectral aliasing. Here, the $fs_{\text{original}}$ refers to the sampling frequency of the raw waveform. The cutoff was set conservatively below the Nyquist frequency.

An 8th-order Butterworth low-pass filter H(z) was applied using zero-phase filtering (filt-filt) to avoid phase distortion. The filtered signal was resampled using polyphase resampling with integer up- and down-sampling factors. For upsampling, a 6th-order Butterworth filter with cutoff $0.9\times fs_{\text{original}}∕2$ removed high-frequency noise before interpolation. The 250 Hz target balances temporal resolution for ECG and PPG morphology and computational efficiency.

Baseline wander was removed only when detected via power spectral analysis. The power spectrum density (PSD), S(f) is computed as follows:

(1) $$S(f)=\underset{T\to \infty }{\text{lim}}\frac{1}{T}{|{\hat{x}}_{T}(f)|}^{2}$$

where ${\hat{x}}_{T}(f)$ is the frequency-domain representation of signal x(t). Power metrics were:

(2) $$P_{<1\text{Hz}}=\sum_{0<f<1}S(f)$$

(3) $$P_{1\text{-}30\text{Hz}}=\sum_{1\leq f\leq 30}S(f)$$

Correction was applied [57] when:

(4) $$\frac{P_{<1\text{Hz}}}{P_{1\text{-}30\text{Hz}}}>\theta_{\text{bw}}$$

Here, the data-driven threshold $\theta_{\text{bw}}$ is empirically selected as 10. When applied, a 4th-order zero-phase high-pass Butterworth filter with cutoff $f_{c}=1.0\;\text{Hz}$ was used. The transfer function of the 4th-order Butterworth high-pass filter is:

(5) $$H(s)=\frac{s^{4}}{s^{4}+2.613s^{3}+3.414s^{2}+2.613s+1}$$

This removes slow drift while preserving cardiac rhythm content (~0.5–5 Hz). The conditional approach keeps signals with minimal drift unchanged.

Powerline noise (fundamental and harmonics) was suppressed via adaptive spectral peak detection and replacement. For fundamental $f_{\text{pl}}$ (50 Hz or 60 Hz, dataset-dependent), the method targets $f_{\text{pl}}$, $2f_{\text{pl}}$, and $3f_{\text{pl}}$ within ±2 Hz. Local statistics used a moving window (0.1 Hz width, minimum 5 samples). After that, Signals were rescaled to millivolts to standardize amplitude distributions. This normalization stabilizes training and prevents codebook collapse. Non-overlapping 10-second segments (2500 samples at 250 Hz) were extracted. Each segment $x_{i}[n]$ was independently normalized by implementing z-score normalization. $\epsilon =10^{-8}$ is used as a denominator to prevent zero division by zero. This removes intersegment amplitude variations while preserving morphology.

To prevent data leakage, the datasets were split into training and validation sets at an early stage of the preprocessing pipeline using a patient-wise partitioning strategy. This ensures that recordings from the same individual do not appear across multiple splits. There is no overlap between the pretraining and evaluation datasets. The dataset from multiple tertiary sites is used exclusively for model pretraining, while the remaining datasets are reserved for assessing the model’s ability to adapt to unseen clinical sites via lightweight fine-tuning.

### CardioFM model architecture

The proposed CardioFM is a self-supervised architecture for multimodal ECG-PPG learning (Figure 1). It includes modality-specific encoders, cross-modal attention, adaptive residual quantization, and modality-specific decoders.

### Input Specification and Dimensional Flow

The model processes paired 10-second physiological signals sampled at 250 Hz, with inputs $x_{\text{ECG}}$, $x_{\text{PPG}}\in R^{B\times 1\times 2500}$, where *B* is the batch size. These are encoded, cross-modally fused, quantized, and decoded to produce reconstructed signals and discrete token representations for downstream use.

### Modality-Specific Encoder Architecture

Each encoder (ECG and PPG) is a 1D CNN that reduces temporal resolution while expanding feature channels. The stem consists of a stride-2 convolution (kernel size 7) followed by a stride-2 max pooling (kernel size 2), reducing the input from 2500 to 625 and expanding to 32 channels. Four residual blocks with stride-2 convolutions progressively expand channels (32 → 64 → 128 → 256 → 512) and reduce temporal resolution (625 → 313 → 157 → 79 → 40). Each residual block implements:

(6) $$y=\text{GELU}({\mathrm{Conv}}_{\text{main}}(x)+{\mathrm{Conv}}_{\text{skip}}(x))$$

where GELU is Gaussian Error Linear Unit, Conv_main_ denotes two 3 × 1 convolutions with batch normalization, and Conv_skip_ is a 1 × 1 strided convolution (Pointwise Convolution) for dimensionality matching. A final 1 × 1 projection maps to *D* = 256, producing latent representations $z_{\text{ECG}}$, $z_{\text{PPG}}\in R^{B\times 256\times 40}$. Encoder weights are initialized using Kaiming normal initialization, and batch normalization parameters are initialized to unit variance.

### Cross-Modal Attention Mechanism

A bidirectional cross-modal attention module with *N*_layers_ = 2 fuses ECG and PPG representations. After transposing to sequence format (*B*, 40, 256), learnable positional embeddings $P\in R^{1\times 40\times 256}$ are added. Each layer includes:

(1) Self-attention per modality:

(7) $${\text{Attn}}_{\text{self}}(z)=\text{softmax}\left(\frac{QK^{T}}{\sqrt{d_{h}}}\right)V$$

where *Q* (Query), *K* (Key), and *V* (Value) are learned vector representations of the input data ($z_{\text{ECG}}$ or $z_{\text{PPG}}$). $Q,K,V\in R^{B\times 40\times 256}$ are linear projections, *d_h_* = 32 is the head dimension, and attention uses *H* = 8 heads with scaling $\sqrt{d_{h}}\approx 5.66$.

(2) Bidirectional cross-attention:

(8) $$z_{\text{ECG}}^{\text{fused}}=z_{\text{ECG}}+\text{CrossAttn}(\text{LN}(z_{\text{ECG}}),\text{LN}(z_{\text{PPG}}),\text{LN}(z_{\text{PPG}}))$$

(9) $$z_{\text{PPG}}^{\text{fused}}=z_{\text{PPG}}+\text{CrossAttn}(\text{LN}(z_{\text{PPG}}),\text{LN}(z_{\text{ECG}}),\text{LN}(z_{\text{ECG}}))$$

where LN is pre-norm layer normalization.

(3) Feed-forward networks with expansion-contraction:

(10) $$\text{FFN}(z)={\text{Linear}}_{2}(\text{Dropout}(\text{GELU}(\text{Dropout}({\text{Linear}}_{1}(z)))))$$

where Linear_1_ : 256 → 1024 and Linear_2_ : 1024 → 256, with dropout *p* = 0.1. Prenorm residual connections are applied throughout. Attention weights use Xavier uniform initialization. The module outputs fused representations $z_{\text{ECG}}^{\text{fused}}$, $z_{\text{PPG}}^{\text{fused}}\in R^{B\times 256\times 40}$.

### Adaptive Residual Vector Quantization

Separate quantizers convert continuous latents into discrete tokens using *L* = 8 stages of residual vector quantization with *K* = 64 codewords per stage (512 total per modality) [58]. Each stage uses a neural codebook generator $f_{\theta_{l}}$ that adapts codebooks based on partial reconstruction. For each temporal position *t*, quantization proceeds iteratively:

(11) $$r_{t}^{\left(l\right)}=z_{t}-{\hat{z}}_{t}^{\left(l-1\right)}$$

The adaptive codebook is generated as:

(12) $$C_{\text{adaptive}}^{\left(l\right)}=C_{\text{base}}^{\left(l\right)}+\delta_{\text{offset}}^{\left(l\right)}$$

where $C_{\text{base}}^{\left(l\right)}\in R^{K\times D}$ is initialized uniformly in [$-\frac{1}{\sqrt{D}},\frac{1}{\sqrt{D}}$], and $\delta_{\text{offset}}^{\left(l\right)}$ is produced by:

(13) $$\delta_{\text{offset}}^{\left(l\right)}=\eta \cdot \mathrm{Tanh}(W_{2}\cdot \text{GELU}(W_{1}\cdot [C_{\text{base}}^{\left(l\right)};c_{\text{cond}}^{\left(l\right)}]))$$

Here, $c_{\text{cond}}^{\left(l\right)}$ comes from a 2-layer Multilayer Perceptron (MLP) with LayerNorm processing ${\hat{z}}_{t}^{\left(l-1\right)}$, [·; ·] denotes concatenation, *η* = 0.1 is the offset scale, and *W*_1_ : 2*D* → *D*, *W*_2_ : *D* → *D* are learnable.

Codeword selection uses nearest-neighbor search via L2 distance:

(14) $$s_{t}^{\left(l\right)}=\underset{k\in \{1,\ldots ,K\}}{\text{arg min}}\;{\left\|r_{t}^{\left(l\right)}-c_{k}^{\left(l\right)}\right\|}_{2}^{2}$$

The partial reconstruction is updated as:

(15) $${\hat{z}}_{t}^{\left(l\right)}={\hat{z}}_{t}^{\left(l-1\right)}+c_{s_{t}^{\left(l\right)}}^{\left(l\right)}$$

After *L* = 8 stages, this yields discrete token indices *s_t_* ∈ {1, … , *K*}^*L*^ and quantized representations $z_{t}^{\text{quant}}\in R^{D}$ for each of *T* = 40 temporal positions.

### Decoder Architecture

Each decoder reconstructs signals via transposed convolutions. An input projection expands 256 → 512 channels. Four residual upsample blocks progressively reduce the number of channels (512 → 256 → 128 → 64 → 32) and upsample temporally (40 → 80 → 160 → 320 → 640). Each upsample block uses:

(16) $$y=\text{GELU}({\text{ConvTranspose}}_{\text{main}}(x)+{\text{ConvTranspose}}_{\text{skip}}(x))$$

Two additional upsampling stages (32 → 32 → 16 channels, 640 → 1280 → 2560 samples) are followed by a final 7 × 1 convolution producing $\hat{x}\in R^{B\times 1\times 2500}$. Decoder weights use Kaiming normal initialization.

### Baseline models

CardioFM was evaluated against five baseline architectures representing conventional and contemporary approaches to physiological time series analysis.

CNN1D comprised four convolutional layers with batch normalization, ReLU activations, and strided max pooling, reducing 2,500-sample inputs to 156 samples before classification via a two-layer MLP. ResNet1D incorporated residual connections through a 7×1 stem convolution followed by four stages with progressive channel expansion (64→512) and skip connections to facilitate gradient flow in deeper architectures [59]. Transformer employed a 4-layer encoder with 256-dimensional embeddings, sinusoidal positional encodings, and multi-head self-attention to capture long-range temporal dependencies across all temporal positions [31]. RVQ and VanillaVQ utilized two-stage pipelines with fixed quantization schemes. RVQ applied 8-stage residual vector quantization with static codebooks, while VanillaVQ employed single-stage quantization. Both extracted 512-dimensional embeddings subsequently classified via MLP heads trained on frozen features.

All baselines were trained on identical data partitions. CardioFM consistently outperformed these architectures, supporting the hypothesis that adaptive codebooks enable context-aware quantization accommodating signal heterogeneity, whereas fixed quantization schemes constrain representational capacity [60].

### Training Pipeline and Implementation

Model training comprises two stages: self-supervised pretraining and task-specific fine-tuning. All implementations used PyTorch on a secure HPC cluster with HIPAA-compliant access controls. De-identified patient waveform data were stored and analyzed within this environment.

#### Pretraining

Pretraining employs a self-supervised reconstruction objective combining signal reconstruction and quantization commitment terms, requiring only modality-level annotations (ECG or PPG) without clinical labels. The total loss per batch is:

(17) $${ℒ}_{\text{total}}=\lambda_{\text{ECG}}{ℒ}_{\text{rec}}^{\text{ECG}}+\lambda_{\text{PPG}}{ℒ}_{\text{rec}}^{\text{PPG}}+\alpha ({ℒ}_{\text{commit}}^{\text{ECG}}+{ℒ}_{\text{commit}}^{\text{PPG}})$$

with λ_ECG_ = λ_PPG_ = 1.0 and *α* = 0.25. The reconstruction loss uses smooth L1 (Huber) loss:

(18) ℒrecmod=SmoothL1(xmod,x^mod)={0.5(x−x^)2∕βif∣x−x^∣<β∣x−x^∣−0.5βotherwise}

The commitment loss regularizes encoder outputs to remain proximal to selected codebook codewords:

(19) $${ℒ}_{\text{commit}}^{\text{mod}}=\frac{1}{B\cdot T\cdot L}\sum_{b=1}^{B}\sum_{t=1}^{T}\sum_{l=1}^{L}{\left\|z_{b,t}^{\text{fused}}-\text{sg}(c_{s_{b,t}^{\left(l\right)}}^{\left(l\right)})\right\|}_{2}^{2}$$

where *B* denotes batch size, *T* = 40 temporal tokens, *L* = 8 quantization stages, $z_{b,t}^{\text{fused}}\in R^{256}$ the fused latent vector, $c_{s_{b,t}^{\left(l\right)}}^{\left(l\right)}\in R^{256}$ the selected codeword at stage *l*, and sg(·) the stop-gradient operator. Gradients propagate via the straight-through estimator:

(20) $$z_{\text{STE}}^{\text{quant}}=z^{\text{fused}}+(z^{\text{quant}}-z^{\text{fused}}).\text{detach}()$$

Here, the detach() operation effectively removes the term from the automatic differentiation gradient tape. This allows the gradient to bypass the non-differentiable quantization step, flowing directly from the quantized output back to the continuous latent representation *z*_fused_, ensuring the encoder remains end-to-end trainable. The model was trained with a per-GPU batch size of 32 (effective batch size 256 across 8 GPUs) using AdamW optimization with parameter-wise weight decay (*λ_w_* = 0.1 for convolutional and linear weights; *λ_w_* = 0 for biases, normalization layers, and positional embeddings). Momentum parameters were *β*_1_ = 0.9, *β*_2_ = 0.999, and *ϵ* = 10^−8^. The learning rate schedule employed cosine annealing with warm restarts. The schedule restarts every 25 epochs, using a maximum learning rate of *η*_max_ = 1.0 × 10^−4^ and a minimum learning rate of *η*_min_ = 1.0 × 10^−6^.

The architecture comprised 8 attention heads, 2 cross-modal attention layers, and a dropout rate of 0.1, totaling approximately 17.11 million trainable parameters distributed across modality-specific encoders, cross-modal attention modules, adaptive quantization generators, decoders, and learnable codebooks. Pretraining was conducted on preprocessed ECG-PPG segments from a multiple tertiary site dataset for 25 epochs using 8 NVIDIA H200 GPUs (512 GB system memory) with PyTorch DistributedDataParallel and NCCL backend, requiring approximately 2 weeks.

### Fine-tuning

For downstream tasks, quantized embeddings were extracted from the frozen pretrained encoder. Each 10-second segment (2,500 samples at 250 Hz) yields embeddings of shape (*B*, 256, 40) per modality. Modality-specific representations are obtained via temporal mean pooling:

(21) $$z_{\text{ECG}}^{\text{pooled}}=\frac{1}{T}\sum_{t=1}^{T}z_{\text{ECG}}^{\text{quantized}}[:,:,t],\;\;z_{\text{PPG}}^{\text{pooled}}=\frac{1}{T}\sum_{t=1}^{T}z_{\text{PPG}}^{\text{quantized}}[:,:,t]$$

yielding (*B*, 256)-dimensional embeddings per modality, subsequently concatenated:

(22) $$z_{\text{combined}}=[z_{\text{ECG}}^{\text{pooled}};z_{\text{PPG}}^{\text{pooled}}]\in R^{B\times 512}$$

The fine-tuning objective employs weighted cross-entropy loss to address class imbalance:

(23) $${ℒ}_{\text{CE}}=-\frac{1}{N}\sum_{i=1}^{N}\sum_{c=1}^{C}w_{c}\cdot y_{i,c}\;\log({\hat{y}}_{i,c}),\;\;w_{c}=\frac{N}{C\cdot N_{c}}$$

where *y_i,c_* ∈ {0, 1} is the ground-truth label, ${\hat{y}}_{i,c}=\text{softmax}{\left(y\right)}_{c}$ the predicted probability, and *N_c_* the number of samples in class *C*. A two-layer MLP classifier maps concatenated features to task-specific outputs. Only MLP parameters are updated during fine-tuning; the pretrained encoder remains frozen, isolating the contribution of learned representations and enabling efficient adaptation with limited labeled data. Optimization used Adam with learning rate *η* = 10^−4^ and batch size 32. Fine-tuning heads add 0.5–2 million parameters depending on task complexity, with per-epoch training time ranging from 5 to 15 minutes.

### Software Environment

Data preprocessing utilized Python libraries including Pandas (≥1.2.0), NumPy (≥1.19.0), SciPy (≥1.6.0) for signal processing, H5Py (≥3.0.0) for HDF5 I/O, WFDB (≥4.1.0) for PhysioNet waveform formats, PyTorch (≥1.9.0), and Matplotlib (≥3.3.0). The modular codebase architecture enables reproducible experiments and straightforward extension to additional downstream tasks.

## Supplementary Material

**Supplementary Information:** The manuscript does not contain a supplementary material file.

## Figures and Tables

**Table 1:** Performance comparison of ECG feature reconstruction across different models. Evaluation metrics include coefficient of determination (R^2^), mean absolute error (MAE in milliseconds for timing features, beats per minute for heart rate), and root mean square error (RMSE). CardioFM consistently demonstrates superior reconstruction accuracy across all electrophysiological parameters.

Model	Heart Rate (BPM)			QT Interval (ms)			QRS Duration (ms)		
	R^2^	MAE	RMSE	R^2^	MAE	RMSE	R^2^	MAE	RMSE
**CardioFM**	**0.865**	**4.006**	**5.01**	**0.77**	**20.219**	**25.56**	**0.853**	**6.718**	**8.33**
ResNet1D	0.653	6.742	8.307	0.613	27.417	34.08	0.782	8.15	10.249
Transformer	0.741	5.669	7.068	0.671	24.475	31.047	0.792	8.073	9.999
RVQ	0.797	4.968	6.204	0.697	23.853	29.7	0.836	7.031	8.818
VanillaVQ	0.694	6.144	7.741	0.734	22.105	27.635	0.628	10.78	13.725
CNN1D	0.729	5.868	7.244	0.659	25.073	31.708	0.674	10.113	12.751

**Table 2:** Performance comparison of PPG feature reconstruction across different models. Evaluation metrics include coefficient of determination (R^2^), MAE in ms for timing features, BPM for pulse rate, and RMSE.

Model	PAT (ms )			T_on-off_ (ms)		
	R^2^	MAE	RMSE	R^2^	MAE	RMSE
**CardioFM**	**0.834**	**22.737**	**28.66**	**0.737**	**59.48**	**74.50**
ResNet1D	0.698	31.478	39.468	0.662	67.31	85.40
Transformer	0.732	29.539	37.004	0.701	63.77	79.81
RVQ	0.742	28.793	36.23	0.628	72.73	90.14
VanillaVQ	0.748	28.672	35.767	0.572	79.02	97.66
CNN1D	0.715	29.967	38.265	0.614	73.34	92.23
Model	Pulse Rate (BPM)			T_sp-sp_ (ms)		
	R^2^	MAE	RMSE	R^2^	MAE	RMSE
**CardioFM**	**0.827**	**6.089**	**7.66**	**0.763**	**55.44**	**70.14**
ResNet1D	0.728	7.836	9.744	0.558	79.42	99.19
Transformer	0.769	7.059	8.940	0.629	71.12	89.70
RVQ	0.795	6.802	8.376	0.704	63.03	79.10
VanillaVQ	0.594	9.667	12.178	0.571	77.89	97.48
CNN1D	0.637	9.146	11.429	0.741	58.98	73.57

## Data Availability

Continuous electrocardiogram (ECG) and photoplethysmography (PPG) waveform data were retrospectively collected from multiple tertiary care hospitals in the United States, encompassing large academic referral centers that provide high-acuity inpatient care. These institutions serve diverse patient populations and include medical, surgical, and cardiac intensive care units, as well as perioperative monitoring environments. The dataset comprises recordings from adult patients admitted for a wide range of clinical conditions, including but not limited to cardiovascular disease, sepsis, respiratory failure, trauma, and postoperative hemodynamic instability. Waveforms were acquired as part of routine clinical monitoring using bedside patient monitoring systems and were de-identified prior to analysis in accordance with institutional policies and applicable data-use agreements. To support robust multimodal representation learning and generalization across care settings, waveform data from these tertiary hospitals were supplemented with ECG–PPG recordings from publicly available datasets representing ambulatory, laboratory, and consumer-wearable environments. All datasets were partitioned at the patient level to ensure that recordings from the same individual did not appear across training, validation, and evaluation splits. Only temporally aligned ECG Lead II and finger or ear PPG waveform segments meeting predefined signal quality criteria were included for model pretraining. No diagnostic labels or outcome annotations were used during the self-supervised pretraining phase.

## Code Availability:

All source code for the CardioFM model architecture, self-supervised pretraining pipeline, downstream fine-tuning heads, and evaluation scripts will be released as an open-source repository at https://github.com/Hassan1503/CardioFM upon acceptance, under the MIT license.
